# Supplementary material for: High Basal Expression and Dual Stress Responsiveness of Soybean (Glycine max) Resistance Gene SRC4
Source: Plants (Basel). 2025 Sep 9;14(18):2820. doi: 10.3390/plants14182820 (PMC12473679; doi:10.3390/plants14182820)
Supplement: Supplementary file 1 [file plants-14-02820-s001.zip › plants-3851478-supplementary.pdf]

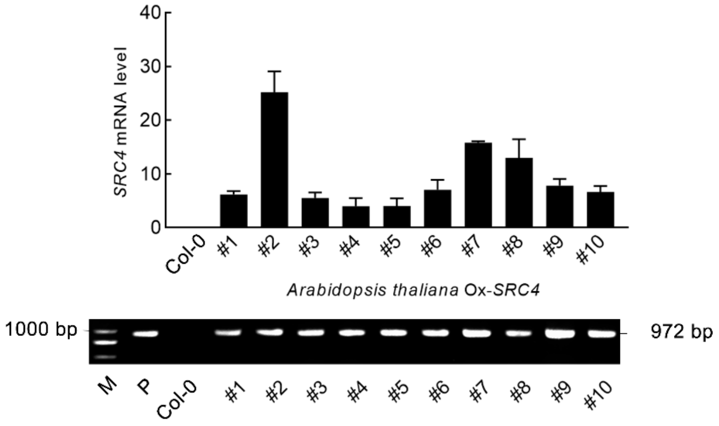

Supplementary Figure S1. Screening and verification of *SRC4* overexpressing transgenic *Arabidopsis* lines. Upper panel: Detection of *SRC4* expression levels in T_2 transgenic *Arabidopsis* lines. Quantitative RT-qPCR analysis was performed on 10 independent Ox-*SRC4* transgenic lines (#1-#10) and wild-type Col-0 to detect relative expression levels of *SRC4* mRNA. Expression was normalized to *Arabidopsis* actin gene. Data represent mean ± standard deviation of three biological replicates. Lower panel: Genomic PCR verification of transgenic *Arabidopsis*. *SRC4*-specific primers were used to amplify genomic DNA from each transgenic line, with amplification product size approximately 972 bp. M: DNA molecular weight standard (Marker); P: positive control; Col-0: wild-type *Arabidopsis* control; #1-#10: independent transgenic lines. PCR products were separated by 1% agarose gel electrophoresis.

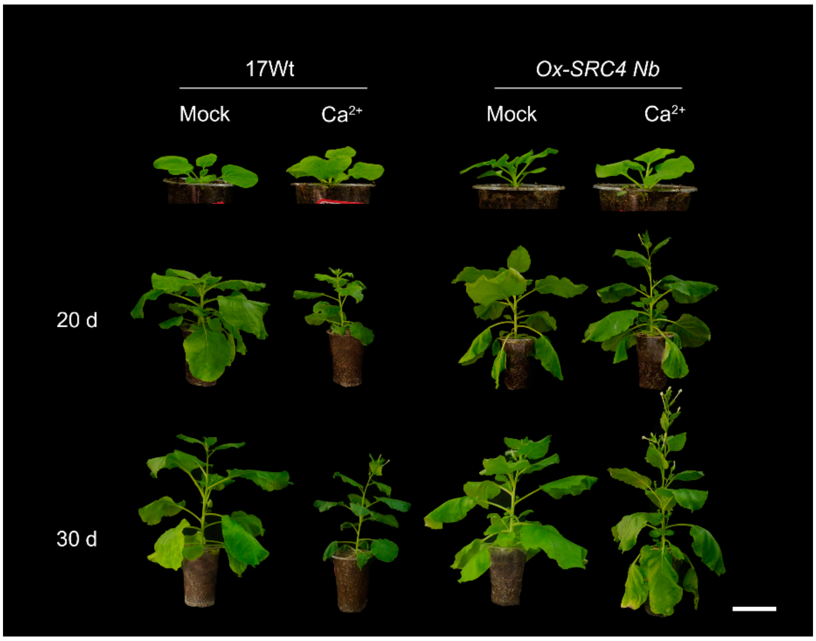

Supplementary Figure S2. Morphological comparison of transgenic tobacco plants stably expressing SRC4 and wild-type controls under different treatment conditions. Representative photographs of wild-type 17Wt and Ox-*SRC4* transgenic tobacco (*Nicotiana benthamiana*) plants grown under mock treatment (ddH_2O) and calcium treatment (Ca^{2+}) conditions. Plants were photographed at 20 and 30 days after treatment initiation. Ox-*SRC4* transgenic lines exhibited enhanced growth performance compared to wild-type controls, with more pronounced differences observed under Ca^{2+} supplementation. Scale bar = 2 cm.
